# Novel ^175^Yb-Poly (L-Lactic Acid) Microspheres for Transarterial Radioembolization of Unrespectable Hepatocellular Carcinoma

**DOI:** 10.22037/ijpr.2019.1100668

**Published:** 2019

**Authors:** Mina Jamre, Mojtaba Shamsaei, Mohammad Ghannadi Maragheh, Sodeh Sadjadi

**Affiliations:** a *Faculty of Energy Engineering and Physics, Amirkabir University of Technology, Tehran, Iran. *; b *Radiation Application Research School, Nuclear Science and Technology Research Institute (NSTRI), Tehran, Iran. *; c *Material and Nuclear Fuel Cycle School, Nuclear Science and Technology Research Institute (NSTRI), Tehran, Iran.*

**Keywords:** Poly (L-lactic acid), Radio microsphere, Ytterbium-175, Tumor therapy, Reactor

## Abstract

Novel biodegradable Poly (L-lactic acid) (PLLA) microspheres containing ytterbium were designed for intra-tumoral radiotherapy, especially for radioembolization. ^175^Yb possess both therapeutic beta and diagnostic gamma radiations. In this work, a process of making ready radiomicrospheres ^175^Yb (acac)_3_-loaded PLLA for more consideration has been investigated. The radiomicrospheres were prepared with approximate size of 20-40 µm, and radionuclidic purity > 92%. The radiomicrospheres were stable *in-vitro* for up to 72 h in normal saline, and also in human serum albumin (HSA). Biodistribution in mice bearing 4T1 tumor showed specific radionuclide uptake over 48 h. Tumor necrosis was also observed at the injection site up to 12 days after injection. These data indicated that ^175^Yb-PLLA microspheres could be prepared and considered further for radiomicrospheres tumor therapy.

## Introduction

In order to reduce the morbidity rate of conventional radiotherapy and to enhance treatment effects on malignant tumors, it is highly desirable to restrict accurately the radiation to the localized tumor area. Among different tumors, hepatocellular carcinoma (HCC) is now the third cause of cancer deaths throughout the world, with over 500,000 people affected (1). The hepatic tumors, including both HCC and metastatic lesions, obtain more than 90% of their blood from the hepatic artery (2). One emerging treatment option for patients with unrespectable liver tumors is therapy of solid tumor that employs radioembolic microspheres of 20-40 µm (3-4).

^90^Y glass-based (Nordion, Canada) and ^90^Y resin-based (SIRTex, Australia) microspheres are commercially available for tumor therapy (5). The major disadvantage of these products is that they are not metabolized and may continue to exist in the target tissue long after radioisotope decay (6). So to make repair for this deficiency, among the several materials suitable for tumor therapy, the biodegradable polymer of lactic acid (PLA) has shown promise because of its chemical stability and biocompatibility (7). The radionuclide ^90^Y a pure beta emitter also has some disadvantages. The distribution of ^90^Y microspheres after each procedure is difficult to verify and even if bremsstrahlung scintigraphy can be done, the image quality is poor. The other drawback for the use of the ^90^Y-microspheres is that ^90^Y is commonly produced in a high cost strontium-90 (^90^Sr) generator or in high flux reactor (^89^Y, has a low neutron cross section of 1.3 b) (8). 

To resolve the above drawbacks, a wide variety of radiomicrospheres have been proposed and quite a few of them have been tested in animal models as well as in humans (9). ^166^Ho (10-14), ^186^Re (6), and ^188^Re (15) can be incorporated into PLLA microspheres. ^175^Yb is one of the potential lanthanides with favorable decay characteristics for developing various radiotherapeutic agents (16-20). ^175^Yb decays by emission of β-particles with 470 keV maximum energy (86.5%) to stable ^175^Lu with a convenient half-life of 4.2 d. ^175^Yb also emits photons of 113 keV (1.9%), 282 keV (3.1%) and 396 keV (6.5%) (21). ^175^Yb can be produced by thermal neutron bombardment of natural ytterbium target. The simplified production scheme is: ^174^Yb (n, γ) ^175^Yb → ^175^Lu (Stable) σ = 69 barn (22), the significantly large thermal neutron capture cross section of ^174^Yb, enables the production of ^175^Yb with much higher specific activity that ultimately results in a higher specific activity of the microsphere formulation. 

The aim of this article was to describe the radiochemistry and analytical assessment of prepared ^175^Yb-PLLA microspheres as a potential agent for tumor therapy and also biological evaluations in a 4T1 xerograph mouse mammary tumor model. Furthermore, the tumor response to radiotherapy treatment was verified using the diagnostic agent ^99m^Tc-Bombesin.

## Experimental

*Materials*

PLLA of molecular weight of 59-101K in chloroform (0.1% w/v; viscosity ~1.0 dl/g), poly(vinyl)alcohol (PVA) of molecular weight 60K, 72K and 146-186K, and ytterbium (III) oxide (natural, 99.9%), Hydrochloric acid, ammonia, acetylacetonate (acac) and dichloromethane (DCM) were supplied by the Aldrich Chemical Company. Sodium pertechnetate (Na^99m^TcO_4_) from a commercial ^99^Mo/^99m^Tc generator and bombesin cold kit were supplied by Pars Isotope Company. The cell culture medium was Roswell Park Memorial Institute (RPMI-1640) supplement with 10% fetal bovine serum (FBS), amino acids, vitamins, and penicillin/streptomycin (Gibco). Mice (BALB/c; male; age 9+1 weeks) and the 4T1 mouse mammary tumor cell line were purchased from Pasteur Institute of Iran.

Fourier transform infrared (FT-IR) spectra were recorded with a Brucker Vector 22 spectrometer (Germany) using KBr disks. Surface morphology and size of particles were investigated by scanning electron microscopy (ZEISS- EVO 18 SEM). Radio thin-layer chromatography (RTLC) was performed on a Raytest-GITA scanner), using silica gel plates (Whatman). Quantitative gamma counting was done for counting distributed activity in mice organs on an EG&G/ORTEC (Model 4001M) NaI (Tl) counter. Also imaging was made at a small area mobile gamma camera (Siemens).

*Preparation of* ^175^ *Yb-PLLA microspheres*

The cold ytterbium (III) acetylacetone (Yb(acac)_3_) from ytterbium (III) oxide (Yb_2_O_3_, natural ytterbium with average molecular weight of 173 g/mol) was prepared according to a literature report (23) with slight modification. Briefly, Yb_2_O_3 _(30 mg) was mixed with hydrochloric acid until complete dissolution. The crude YbCl_3_ solution was then mixed with the second solution containing acethylacetone in aqueous ammonia at pH=7 (v: v = 1:3). A white precipitate appeared on slow addition of aqueous ammonia. Yb(acac)_3_ crystals were obtained by filtration over a 5.0 μm Millipore paper filter, washed with water (3x 5 mL) and air dried at the room temperature. The preparation of Yb(acac)_3_ were examined by FT-IR. In the following Yb-PLLA microspheres were acquired through process consists of dissolving Yb(acac)_3_ (20-60 mg) in the organic solvent DCM (0.5 mL) containing PLLA polymer (12 mg). Then organic phase was poured slowly with a syringe into a beaker containing an aqueous solution of PVA (concentration (2-3%; 2-10 mL). With mechanical stirring (800-1200 rpm) the organic solvent was evaporated. Afterwards, the aqueous suspension was centrifuged (2000 rpm; 10 min), and the solid pellet was washed with water (5x 5 mL). The solid cold Yb-PLLA microspheres were freeze-dried and its characteristics consist of surface morphology and sizes of particles were defined by scanning electron microscopy (SEM).

The radioactive ^175^Yb-PLLA microspheres were achieved by neutron irradiation of cold Yb-PLLA microspheres (40 mg) after packing in quartz ampoule and irradiation by neutron (flux 4×10^12 ^n cm^-2^ s^-1^) for 4 h at the reactor. The target was subsequently cooled for 48 h to eliminate the possibility of the presence of radionuclides impurity. Radiochemical processing was performed by dispersing the irradiated target in water inside a lead-shielded plant. The amount of activity produced, radionuclidic purity and morphology analysis were determined for prepared radiomicrospheres.

**Table 1:** Optimization of different parameters for the formation of microspheres

**PVA molecular weight**	**PVA concentration (% w/v)**	**O/W(v/v)**	**Formation of microspheres and surface morphology**
60K	2%	1:20	Not formed
72K	2%	1:20	Not formed
146-186K	2%	1:20	Formed but no smooth surface
146-186K	2%	1:4	Not formed
146-186K	2%	1:10	Not formed
146-186K	3%	1:20	Formed with smooth surface

* PLLA: (10 mg), Yb(acac) : (40 mg), Stirring speed: 1200 (rpm), Solvent: DCM

**Figure 1:**
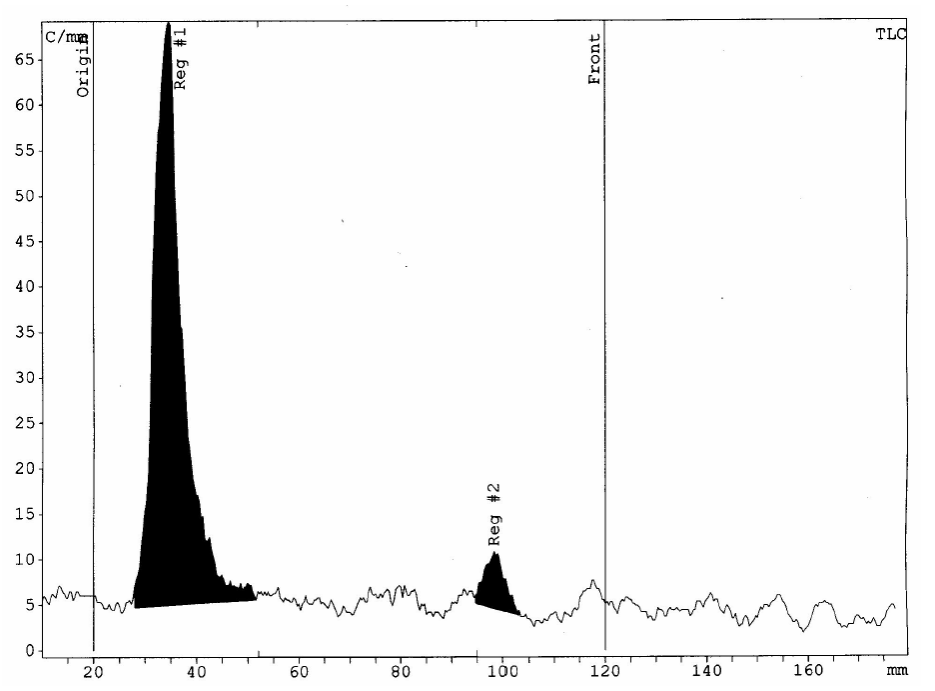
Radiochemical purity of 175Yb-PLLA microspheres solution determined by the RTLC scanner

**Figure 2:**
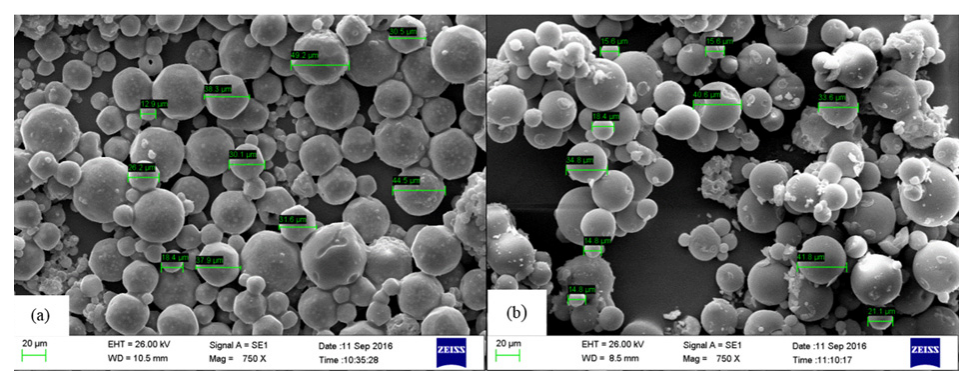
SEM images of Yb-PLLA microspheres (a) before and (b) after 4 h neutron activation, respectively

**Figure 3:**
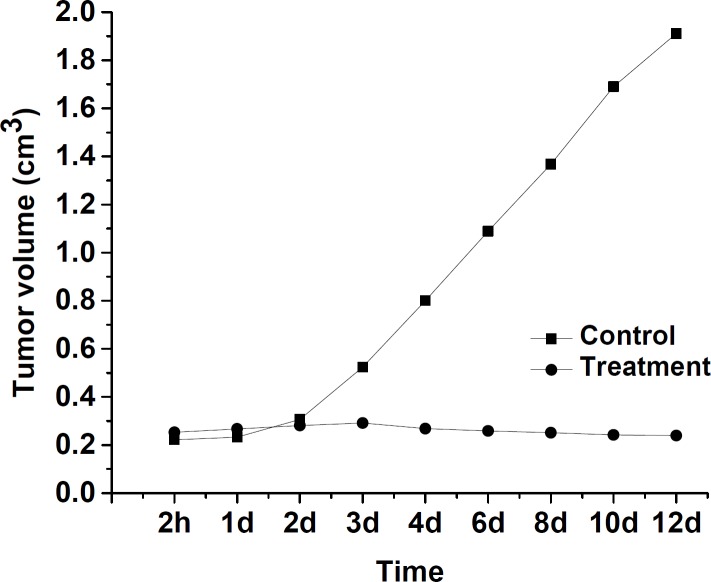
Tumor volumetric assessment of the effect of ^175^Yb-PLLA microspheres in mice

**Figure 4:**
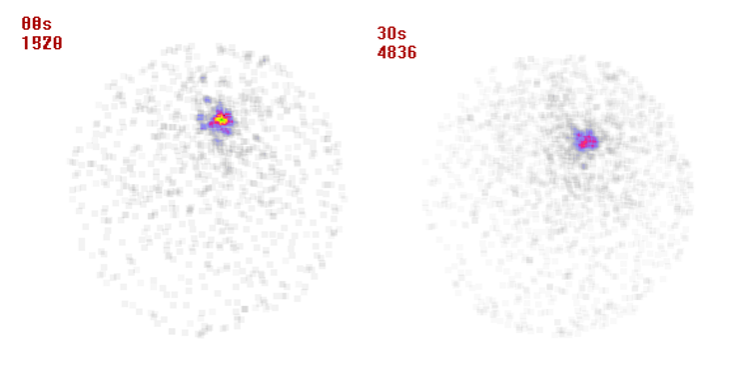
Scintigraphy image of BALB/c mice bearing 4T1 tumor 24 & 48 h after injection of 175Yb-PLLA microspheres, respectively from left to right

**Figure 5:**
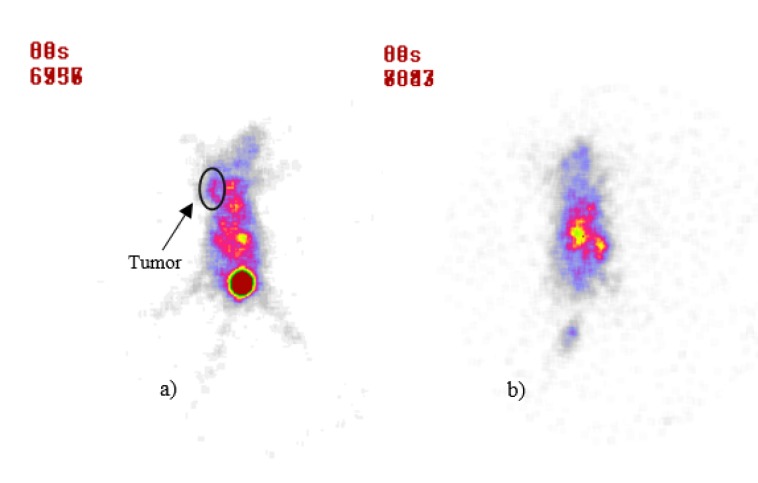
Scintigraphy image of BALB/c mice after 30 min post-injection of 99mTc-Bombesin, (a) control group, (b) treatment group after 12 d post-injection of 175Yb-PLLA microspheres

*Radiochemical analysis and quality control*

The amount of ^175^Yb activity produced and radionuclidic purity of the produced ^175^Yb were determined by counting on an HPGe detector coupled to a Canberra TM multichannel analyzer for 1000 s. The radiochemical purity of the ^175^Yb-PLLA microspheres was verified by using the RTLC method on silica gel 60 as the stationary phase, developed in a mobile phase of aqueous DTPA solution (10 mM; pH=5), to discriminate free ^175^Yb (*R*_f_ = 1.0) from the radiomicrosphere (*R*_f_ = 0.0). The radioactivity was quantified by TLC scanner. SEM analysis of the radiomicrospheres was performed after decay for one-month storage at room temperature in closed vials.

*In-vitro* stability of radiomicrospheres was checked according to ITLC method using a sample of active microsphers stored in NaCl 0.9% (W/V) and in human serum at room temperature during 1, 4, 24, 48 and 72 h post activation. Sampling was done at different time periods after incubation with the volume of 5 µL and after ascending chromatography, plates were scanned for activity.

*Animal studies*

Animal experiments were performed in compliance with the regulations of our institution and with generally accepted guidelines governing such work. A suspension of 4T1 mouse mammary tumor cells in PBS buffer was subcutaneously injected into the left armpit of each BALB/c mouse. A total of 24 tumor bearing nude mice were randomly divided into 2 groups and were used to evaluate the efficacy of treatment. Injections of microspheres were down into the centers of the tumors while the volume of tumors reached approximately 0.2 cm^3^ (estimation of tumor volume using caliper). 

The treatment group (n = 9) was injected intra-tumorally with ^175^Yb-PLLA microspheres (20 MBq; 0.1 mL) and the control group (n = 9) was injected with the same amount of cold Yb-PLLA microspheres. The animals were monitored for the effects on tumor size and body weight for over the duration of 12 days or until the animals reached the maximum allowable tumor burden. The effects of ^175^Yb-PLLA microspheres on were evaluated by comparing the tumor size with the control group.

To determine the tissue radioactivity and the stability of ^175^Yb-PLLA microspheres biodistribution and imaging study were performed for treatment group. For biodistribution after 24 and 48 h, the mice (3 animals for each time point) were sacrificed and activity associated with each organ was measured in a NaI (Tl) scintillation counter. The distribution of the activity in different organs was calculated as the percentage of injected activity (dose) (%ID) per organ (g). Scintigraphy imaging at these time points post-injection also was carried.

Furthermore, to evaluate the viability and biologic activity of the tumor, imaging with radiotracer ^99m^Tc-bombesin was preformed. For this purpose, 20 MBq ^99m^Tc-bombesin in 0.1 mL volume was injected via the femoral vein at 12 days after tumor treatment. At 30 min after injection the mice were anesthetized with 0.01 mL ketamine 20% and 0.10 mL xylazine 3% intra peritoneal and accumulation of the radiotracer in the tumor was assessed by planar scintigraphy using the single head gamma camera (small area mobile, Siemens, 140 keV high sensitivity parallel whole collimator and 15% window around 140 keV). The images were analyzed visually. 

## Results and Discussion

*Preparation of radiomicrospheres*

Tumor targeted biodegradable Poly (L-lactic acid) (PLLA) radiomicrospheres containing beta emitter radionuclide ytterbium-175 were designed and prepared after neutron irradiation of cold Yb-PLLA microspheres. In order to obtain stable radiomicrospheres in a good yield, combination of individual ingredients including Yb (acac)_3_ as a radioligand and PLLA as a polymer agent should be considered. Yb (acac)_3_ was prepared as a white powder in high yield (> 90%). 

The synthesized complex was identified by infrared spectroscopy (FT-IR) and characteristic peaks of 2990 cm^−1^ (aliphatic C-H) and 1730 cm^−1^ (C = O), 1267 cm^−1^, 1145 cm^−1^, and 1140 cm^−1^ (C-O). A wide band was also observed at 3398 cm^-1 ^in FT-IR spectrum of Yb (acac)_3_, which is characteristic for crystallized water.

Yb-PLLA microspheres prepared though oil-in-water (O/W) emulsion solvent extraction procedure by addition of Yb (acac)_3 _and PLLA in DCM as an organic phase to aqueous phase containing PVA. Microspheres formation could be affected in particular by the volumetric ratio of organic (O) and aqueous (W) phases, molecular weight and stabilizer concentration, amount of metal and stirring speed during formation process (Table 1). As Table 1 showed, spheroid and smaller sphere were formed while the volume of the aqueous phase from 1:4 to 1:20 increased. In low volume of aqueous phase (O/W = 1:4) droplet breakdown was reduced and larger microspheres were formed. Furthermore, these findings may be explained on the basis of change in the viscosity of the emulsion formed during processing, which in turn can change the microsphere size and shape.

PVA as a stabilizer was found to be important in microsphere formation. Higher molecular weight of the polymer resulted in the higher viscosity and better emulsion stability and provided highly spherical particles with a smooth surface. Since the main function of PVA is as a stabilizer and a surfactant agent, increasing the molecular weight of the PVA did not affect the microsphere size. 

Effect of employing a higher concentration of PVA could cause formation of smaller particles due to the formation of the tighter micelles around the PLLA microspheres. The droplets were forced to be further apart from each other and became easier to breakdown.

Another important parameter especially related to specific activity was Yb (acac)_3 _complex concentration. When Yb (acac)_3 _complex concentration in the organic phase increased, PVA still formed micelles surrounding the organic phase droplets. However, it did not have the ability to tighten the micelles and did not successfully separate them. Therefore, this resulted in larger, irregular, and agglomerated microspheres.

In different stirring speeds (800, 1000, and 1200 rpm) size of microspheres decreased with increasing stirring speed. It could be explained that at the higher stirring (1200 rpm), the particles were stirred more powerfully which gave more shear force to break and separate the polymer droplets apart. Therefore, the size of the microspheres was reduced.So, these results showed that by using solvent to water volume ratio (1:20), PVA molecular weight (146-186K), PVA concentration (3%), type of solvent (DCM) and stirring speed (1200 rpm) with final adjustment of the Yb (acac)_3_ (40 mg) and PLLA (10 mg) (ratio of 4:1) conduced the preparation of cold Yb-PLLA microspheres (40 mg) with suitable size (20-40 µm). The radiomicrospheres ^175^Yb-PLLA were prepared from different batches having specific activities in the range between 23.4 MBq/mg and 30.1 MBq/mg. The radioactive concentration of the processed ^175^Yb-PLLA microspheres was maintained between 0.9 GBq/mL and 1.2 GBq/mL while the pH was adjusted between 6 and 7. To avoid undesired side-product which increases impurity and inversely affect specific activity yield, use of excessive amounts of Yb-complex and long period of neutron irradiation should be evaded. The labeling efficiency of more than 95% can be achieved by using the optimal amount of Yb-PLLA microspheres (40 mg) for a period of 4 h neutron irradiation. 

*Radiochemical analysis and quality control*

The radionuclidic purity of ^175^Yb as determined by gamma ray spectrometry (^175^Yb; 113, 286, and 396 keV) and was found to be > 92% at 48 h after irradiation. By recording the gamma ray spectra of the sample aliquot, the observed photo peaks correspond to the ^169^Yb (63, 110, 130, 177,198, 261, and 307 keV), and ^177^Lu (208 and 112 keV) were found to be the radionuclidic impurities in the processed ^175^Yb. The radiochemical purity of radiomicrospheres determined by radio thin layer chromatography (RTLC) while aqueous DTPA solution (10 mM; pH=5) was used as solvent to elute the radiomicrosphere complex in a stationery phase. The ^175^Yb^3+^ moved with the solvent front (R_f_ = 1.0) and ^175^Yb-PLLA-microspheres remained at the origin (R_f _= 0.0). In RTLC system radiochemical purity of > 95% and a specific activity of 25.2 MBq/mg for radiomicrospheres was achieved. Small amounts of ^175^Yb (<5.0%) was observed. RTLC results interpretation showed the presence of a single major peak at R_f _= 0 which was related to radiomicrospheres and confirmed its high radiochemical purity. Results prove the absence of significant contamination due to free ^175^Yb in radiomicrospheres (Figure 1).

Effect of neutron activation on radiomicrospheres morphology was evaluated by SEM imaging analysis. Results showed that there was no significant change in morphology and size of microspheres before and after neutron irradiation process (Figure 2). Optimization of activation condition and formulation including the amount of utilized materials lead to preparation of a stable compound with high activation yield. 

Stability of ^175^Yb-PLLA microspheres in saline solution and HSA was investigated. Results showed that labeled microspheres was stable (>99%) up to 72 h after preparation. Significant release of radioactivity or decomposition of microspheres was not observed. The stability may be attributed to the presence of a lot of trap state in the PLLA strings which are used in the structure of the microspheres.

*Animal study*

Tumor volumes study results of radiomicrospheres in the treatment and control groups of BALB/c mouse have been shown in Figure 3. There was no observed discomfort or aberrant behavior in two studied groups. The tumor volume in the control group increased from 0.30 ± 0.01 cm^3^ to 1.91 ± 0.01 cm^3^ up to 12 days of the treatment period. In the treatment group which received ^175^Yb-PLLA-microspheres, the tumor volume change was negligible (0.35 ±0.01 cm^3^ on the first day versus 0.31 ± 0.01 cm^3^ at 12 day later). This low change tumor values indicate that a tumor cellular degeneration has occurred due to beta-irradiation of ^176^Yb in the microspheres which correlate with the amount of microspheres injected activity, radiochemical stability, duration of treatment, and delivered dose received by the tumor in the *in-vivo* environment.

Distribution results of radiomicrospheres have been considered in based on percentage of total activity accumulated in gram of organs (%ID/g). The blood uptake value was 0.04 ± 0.01% ID/g at 24 h post injection which decreased to 0.02 ± 0.01% ID/g at 48 h after injection. Uptake value in kidneys at 24 h after injection was 0.41 ± 0.03% ID/g that reduced to 0.13 ± 0.02% ID/g at 48 h. Uptake values for liver and intestine were 0.67 ± 0.04% ID/g, and 0.10 ± 0.02% ID/g at 24 h and these values reduced to 0.21 ± 0.06% ID/g and 0.05 ± 0.01% ID/g at 48 h, respectively. Results demonstrated fast clearance from blood flow followed by renal and liver excretion. However, very low uptake values of radiomicrospheres in these organs could be related to the stability in their structure as well as their appropriate particle size which prevents them from entering the blood stream. The tumor showed uptake value of 45.78 ± 2.15% ID/g at 24 h which was decreased to 43.40 ± 1.58% ID/g up to 48 h. This negligible decrease demonstrates its reasonable tumor retention and that this tumor retention activity is related to stability of radiomicrospheres.

The tumor location could be visualized through scintigraphy within 24 and 48 h after injection which confirms the specific retention of activity by the tumor (Figure 4). Whole body imaging showed that the radiomicrospheres were concentrated in the tumor in different observation period, and no accumulation of radioactivity was noted in the other tissues.

For detecting apoptosis following ^175^Yb-PLLA microspheres tumor therapy, ^99m^Tc-Bombesin scintigraphy a potential radiotracer for tumor diagnosis was performed. Typical scintigrams at 30 min post-injection of radiotracer for control and treatment groups in 12 d after treatment were illustrated in Figure 5. Compared to control group, radiotracer uptake in tumor was decreased after therapy in treatment group. While the tumor site was well recognizable for the control group, this site was not well recognized for the treatment group. 

The lack of accumulation of radiotracer in the tumor site could be due to necrosis and inactivation of the tumor by radiomicrospheres irradiation. The use of radioactive microspheres administered arterially or itratumorally has the potential to overcome the disadvantages of external beam radiation, which is limited by the radiosensitivity of the healthy tissue. As an alternative for the ^90^Y microspheres which are currently available for internal tumor therapy, in this study a straightforward method for the production of ^175^Yb-PLLA microspheres has been developed (24-26). These microspheres are advantageous in that they combine biocompatibility and low density with the favorable physical characteristics of ^175^Yb, thus enabling image-guided radionuclide therapy. The biodegradability of the poly lactic acid microspheres and their low density (1.4 g/mL) add extra value since it allows for repeated injections and diminishes the chance of settling during administration. Combined with the imageable gamma emission and low production costs of ^175^Yb these microspheres offer an attractive alternative for ^90^Y microspheres and warrant further research in this field.

These data confirm that above designed radiomicrospheres have high stability, uniform size and can be used for tumor therapy. However, for better tumor therapy response, the idea to design radiomicrospheres with higher specific activity, higher stability and release of activity to less degree in *in-vivo* seems highly valuable. To achieve this goal, the replacement of natural ytterbium by enrichment ytterbium (^174^Yb) and use of post labeling method through active ytterbium solution (^175^YbCl_3_) and PLLA polymer with different stabilizer instead of pre labeling method (irradiation of cold Yb-PLLA microspheres) is suggested and these are the objectives of our future studies. 

## Conclusions

The feasibility study has demonstrated novel radiomicrospheres prepared using biodegradable PLLA, loaded with ^175^Yb produced via neutron activation in a nuclear reactor. The ^175^Yb-PLLA microspheres (approximate size of 20–40 μm) were easy to prepare by solvent evaporation technique and the procedure did not involve unnecessary radiation exposure during the process. The loaded microspheres were showed to remain stable and separated while keeping out their spherical shape at the lower neutron flux of 4×10^12^ n cm^-2^ s^-1^ (4 h).^ 175^Yb-PLLA microspheres could be prepared in high yield (23.4 ± 12.12 MBq/mg) with excellent radiochemical purity (> 95%). The microspheres were showed excellent the *in-vitro* stability at room temperature. Biological studies in BALB/c mice bearing 4T1 tumor, showed complete retention of injected radioactivity of the tumor for treatment group within 48 h post-injection, the increasing tumor volume was stopped from the 4th day post-injection and tumor necrosis was visible at the site of injection on the 12^th^ following ^175^Yb-PLLA microspheres administration in comparison with control group scan. These studies demonstrated that the ^175^Yb-PLLA microspheres offer potential as a suitable agent for local intra-tumoral radiotherapy, especially for radioembolization of hepatocellular carcinoma. Dosimetric studies to estimate total ^175^Yb activity were needed to deliver equivalent tumor dose and therapeutic response shall be carried out.

The new radiomicrospheres as a tumor therapy agent which were labeled with ^175^Yb, clearly showed a good specific activity yield (23.4 MBq/mg) with high radiochemical purity (> 95%). Furthermore, these radiomicrospheres displayed suitable stability in human serum without considerable contamination which was confirmed by paper thin layer chromatography. High stability followed by specific tumor retention and inhibition of tumor growth due to the dose transferred to it are important characteristic of these radiomicrospheres. Based on the observed properties, through more complementary studies, these radiomicrospheres could be used as a new treatment agent in the field of intera- tumoral therapy. 
